# Clinical predictors of seizure threshold in electroconvulsive therapy: a prospective study

**DOI:** 10.1007/s00406-012-0342-7

**Published:** 2012-07-15

**Authors:** Jeroen A. van Waarde, Lucas J. B. van Oudheusden, Bastiaan Verwey, Erik J. Giltay, Rose C. van der Mast

**Affiliations:** 1 Department of Psychiatry, Rijnstate Hospital, P.O. Box 9555, 6800 TA Arnhem, The Netherlands; 2 Department of Psychiatry, Leiden University Medical Center, Leiden, The Netherlands

**Keywords:** ECT, (Initial) seizure threshold, Predictors, Prospective study

## Abstract

At the start and during the course of electroconvulsive therapy (ECT), estimation of the seizure threshold (ST) is useful in weighing the expected effectiveness against the risks of side effects. Therefore, this study explores clinical factors predicting initial ST (IST) and levels of ST during the ECT course. This prospective observational study included patients aged ≥18 years receiving ECT without contraindications for dose titration. At the first and every sixth consecutive ECT session, ST level was measured. Using multivariate linear regression and multilevel models, predictors for IST and change in ST levels were examined. A total of 91 patients (mean age, 59.1 ± 15.0 years; 37 % male; 97 % diagnosis of depression) were included. In multivariable analysis, higher age (β = 0.24; *P* = 0.03) and bifrontotemporal (BL) electrode placement (β = 0.42; *P* < 0.001) were independent predictors for higher IST, explaining 49 % of its variation. Also, these two variables independently predicted higher ST levels at different time points during the course. Using multilevel models, absence of a previous ECT course(s) predicted a steeper rise in ST during the course (*P* = 0.03 for the interaction term time*previous ECT). The age-adjusted dose-titration method is somewhat crude, resulting in some measurement error. Concomitant medication use could have influenced ST levels. Increasing age and BL electrode placement predicted higher (I)ST, which should be taken into account when selecting ECT dosage. Previous ECT course(s) may avoid an increase in ST during the course of ECT.

## Introduction

Electroconvulsive therapy (ECT) is an effective and safe treatment for depression as well as for other severe psychiatric conditions, such as catatonia, malignant neuroleptic syndrome, intractable psychosis, mania, and some specific forms of delirium [1, 31]. To be effective, ECT must elicit generalized seizure activity in the brain by using an electrical stimulus that exceeds the seizure threshold (ST) [17]. Although controversy exists about the optimal electrical stimulus dosing strategy [2, 13], there is some consensus that, especially in right unilateral (RUL), the stimulus should substantially exceed the ST [1, 3, 27]. The initial ST (IST) has been defined as the smallest electrical dose needed to produce a generalized seizure of at least 25–30 s on an electroencephalogram (EEG) at the first session [1, 8]. IST is reported to vary substantially between patients [2], although prospective studies are sparse [4, 32].

Higher IST is associated with male gender [4, 21], advanced age [4, 21, 24], bifrontotemporal (BL) electrode placement [4, 10, 18], lower dynamic impedance [10, 23], a greater burden of medical illness [4], higher body mass index (BMI) [4, 7], and previous ECT course(s) and concomitant medication use [1, 4].

Because, on the one hand, the extent of the electrical dose above the IST is associated with more short-term cognitive side effects [26] and, on the other, subconvulsive stimulus administration is related to cardiac arrhythmia [19], better knowledge of the factors determining the individual IST would be clinically useful in weighing the expected effectiveness against the risks of side effects of ECT.

Over a complete treatment course, 40–100 % of patients are reported to show a rise in ST, and failure to reach a substantial ST increase may be a marker of lack of efficacy [9, 22]. Moreover, in RUL ECT, a rise in ST during the course is associated with a significantly lower likelihood of therapeutic response; in addition, older patients are more likely to show ST increase and to be a non-responder [15]. Apart from age, seizure intensity on EEG, total course duration, and electrode placement are suggested to determine a rise in ST [9, 15, 22]. However, those disagreeing with these findings estimate that only 20–46 % of patients would show a rise in ST during the course without negative consequences for the effectiveness of the treatment [1, 11, 28].

Hypotheses to explain the variation in IST and the change in ST during the ECT course in relation to clinical characteristics are sparse [33]. Clinically, it will be helpful if patient characteristics could guide an optimal decision regarding the electrical stimulus, both at the start and during the course of ECT. Therefore, this study is a prospective investigation of clinical factors predicting IST and change in ST during the course of ECT.

## Methods

### Participants

All consecutive patients (from December 1, 2009, to November 15, 2011) with an indication for ECT at the Rijnstate Hospital (a 36-bed psychiatric facility in Arnhem, with a catchment area of 600,000 inhabitants) were asked to participate in this prospective observational study. Patients were excluded if age was <18 years (*n* = 0), no written informed consent was provided (*n* = 5), and contraindications existed for dose titration (e.g., life-threatening condition of the patient, severe cardiovascular comorbidity; *n* = 18). The local medical ethical committee approved the research protocol (trial registration number: NL24697.091.09). Written informed consent was obtained from all participants.

Age, gender, psychiatric disorder(s) classified according to the Diagnostic and Statistical Manual of Mental Disorders (DSM-IV-R) by at least two experienced psychiatrists, somatic comorbidity, BMI (weight/[height]^2^), previous ECT treatment (including time interval between last ECT session and current titration session), and concomitant medication use were documented in a standardized way. Of those patients who did not agree to participate or who were excluded, age, gender, psychiatric diagnoses (DSM-IV-R, axis 1 and 2), and somatic comorbidity were registered. Only patients who were diagnosed with a well-documented personality disorder or who were clinically suspected to suffer from comorbid personality disorder were classified according to the DSM-IV-R, axis 2.

### Instruments

In the week before the first ECT (baseline), severity of the mood disorder was assessed with the Montgomery–Åsberg Depression Rating Scale (MADRS), a validated observer-rated scale that contains 10 items (scored 1–6 per item). Maximum score is 60, with higher scores indicating more and a higher severity of depressive symptoms [20].

Cognitive functioning in the week before the first ECT (baseline) was measured using the Mini-Mental State Examination (MMSE). This validated instrument is commonly used to evaluate cognitive functioning in patients (maximum score is 30, with lower scores indicating more cognitive dysfunction) [12].

Amount and severity of somatic comorbidity were rated using a modified Cumulative Illness Rating Scale (CIRS), consisting of 14 aspects of possible pathology and impairment of major organ systems, which are rated from 1 (no impairment to that organ/system) to 5 (impairment is life threatening; treatment is urgent or of no avail; prognosis is grave), whose sum score ranges from 14 through 70 points [16]. The item “Psychiatric/Behavioral (includes depression, anxiety, agitation, psychosis, not dementia)” was set at 5.

### Electroconvulsive therapy and measurement seizure thresholds

ECT was administered using a constant-current (0.9 Ampère), brief-pulse (0.25 ms [ms] in RUL ECT; 0.5 ms in BL ECT) device (Thymatron IV; Somatics Incorporation, Lake Bluff, Illinois, USA), after intravenous induction of anesthesia with etomidate (1.5 mg/kg body mass), muscle paralysis with succinylcholine (0.5–1 mg/kg body mass), and appropriate oxygenation (100 % oxygen, positive pressure) until the resumption of spontaneous respiration. Electrode placement was initially RUL, except in patients at high risk of suicidality and/or somatic complications, or if previous ECT had successfully been administered bilaterally. The decision as to whether a patient should be switched to BL during the course was based on the clinical judgment of the treating psychiatrist (mostly when less/no clinical response had been reached after six RUL sessions). Treatment parameters (electrode placement, dynamic impedance, etomidate and succinylcholine dose, switch from RUL to BL during the course, and total ECT sessions of the index ECT course) were documented.

ST was measured by an internationally accepted, empirical, age-adjusted, titration method at the 1st (IST), 6th, 12th, 18th, and 24th (ST_6–12–18–24_, respectively) ECT session [3]. Consecutive measurements of ST depended on the duration of the ECT course. Index ECT was terminated if the patient had recovered, showed no (further) clinical improvement over a period of 2 weeks, or had shown no improvement at all after 10 BL sessions, based on the judgment of at least two experienced psychiatrists. If the starting stimulus dose failed to elicit a seizure of at least 20 s of motor activity measured with the cuff method and/or ≥25 s on EEG, stimulus charge was increased according to the titration schedule (for patients aged <50 years: 25.2, 50.4, 100.8, 201.6, and 403.2 mC; for patients aged ≥50 years: 50.4, 100.8, 201.6, and 403.2 mC), and the patient was restimulated after 30 s. After the titration session, dosage was set at 2.5 times the ST for BL and at six times the ST for RUL treatment. Patients were treated twice weekly. During the titration session, if the ST was reached and muscle relaxation and anesthesia were still sufficient, the patient was restimulated at a therapeutic dose in that same session.

### Statistical analyses

Data are presented as means, standard deviations (SD) or standard errors (SE), medians and interquartile ranges (IQR), or numbers and percentages, when appropriate. The excluded patients were compared with the study group using *t* tests for age, MADRS score, and CIRS score, with the Χ^2^-test for gender, and the Mann–Whitney *U* test for the skewed MMSE score. First, descriptive statistics were applied on patient and treatment parameters. Differences between levels of IST and ST_6–12–18–24_ were calculated, and the median change in ST value per ST measurement was compared in the RUL- and BL-treated groups, using Mann–Whitney *U* tests. Second, to detect independent clinical factors predicting IST, univariate regression analyses were performed for all variables, followed by multivariate regression analyses selecting those variables that were associated with *P* values <0.10 in the univariate analyses. Finally, multivariate multilevel regression analyses (i.e., linear mixed models, adjusted for gender, age, CIRS score, MMSE score, previous ECT, electrode placement, dynamic impedance, and succinylcholine dose) were performed to detect predictors of ST levels during the ECT course. Multilevel regression analysis was also used to analyze the association between baseline predictors and changes over time in ST (using appropriate interaction terms of the predictor*time; in which time was used as a continuous variable). In multilevel analyses, we used a compound symmetry covariance model with a two-level structure consisting of up to five observations in time (lower level) and the subject (higher level).

All tests were two-sided with *P* < 0.05 denoting statistical significance. SPSS for Windows version 17.0 (SPSS Inc., Chicago, Illinois, USA) was used for all analyses.

## Results

### Patient and treatment characteristics

Of the initial 114 patients, 91 indicated for ECT were included in this study. Excluded patients (no informed consent [*n* = 5], contraindications for dose titration [*n* = 18]) did not differ from the study group regarding mean age (*t* = −1.436; d*f* = 112; *P* = 0.15), gender (Fisher’s exact test = 0.053; d*f* = 1; *P* = 0.51), mean MADRS score (*t* = 0.445; d*f* = 99; *P* = 0.66), median MMSE score (Mann–Whitney *U* test = 344.5; *Z* = −1.88; *P* = 0.06), and mean CIRS score (*t* = 1.671; d*f* = 112; *P* = 0.10). In the excluded group, there were more patients with a diagnosis of catatonia (Χ^2^ = 12.48; d*f* = 3; *P* = 0.006).

Table 1 shows the patient and treatment characteristics. Mean age was 59.1 ± 15.0 years, 37 % (*n* = 34) was male, nearly all patients (97 %; *n* = 88) were depressed, and 26 % (*n* = 24) of them also had psychotic features. At baseline, mean MADRS score was 36.2 ± 8.6 and median MMSE score was 28 (IQR: 25–29). The CIRS showed a mean score of 22.2 ± 3.8, and higher CIRS scores were significantly related to higher age (*r* = 0.48; *P* < 0.001). Of all patients, 31 % (*n* = 28) had undergone previous ECT course(s), with a median of 646 (IQR: 202–1,393) days between the last previous ECT session and the IST session.

**Table 1:** Patient and treatment characteristics (*n* = 91)

	*n* (%); or mean ± SD^d^; or median; IQR^e^
*Patient characteristics*	
Male gender	34 (37.4)
Age, in years	59.1 ± 15.0
Body mass index, in kg/m^2^	24.7; 22.7–27.3
CIRS score^a^	22.0 ± 3.8
Diagnostic category, according to axis 1 of DSM-IV-R^c^:	
Depressive disorder without psychotic features	50 (54.9)
Depressive disorder with psychotic features	24 (26.4)
Bipolar disorder, depressive episode	14 (15.4)
Other disorders^b^	3 (3.3)
Documented or clinically suspected personality disorder present, according to axis 2 of DSM-IV-R	27 (29.7)
MADRS^f^ score at baseline (*n* = 88)	36.2 ± 8.6
MMSE^g^ score at baseline (*n* = 86)	28; 25–29
*Treatment characteristics*	
Had previous course(s) of ECT	28 (30.8)
Days between last ECT and index ECT (*n* = 28)	646; 202–1,393
Use of seizure threshold influencing concomitant pharmacotherapy:	
Benzodiazepines	54 (59.3)
Antiepileptics	7 (7.7)
Antidepressants	52 (57.1)
Antipsychotics	58 (63.7)
Electrode placement is bifrontotemporal at first titration session	28 (30.8)
Initial seizure threshold, in millicoulombs	60.9 ± 42.3
Dynamic impedance at first titration session, in Ω	275.5 ± 59.5
Etomidate dose at first titration session, in mg	20; 18–22
Succinylcholine dose at first titration session, in mg	80; 75–100
Total number of ECT sessions^h^ (*n* = 86)	17.9 ± 7.4
Switch from right unilateral to bifrontotemporal electrode placement during course^i^ (*n* = 63)	33 (52.4)

^a^Cumulative Illness Rating Scale

^b^Schizophrenia (*n* = 2) and malignant neuroleptic syndrome (*n* = 1)

^c^Diagnostic and Statistical Manual of Mental Disorders

^d^Standard deviation

^e^Interquartile range

^f ^Montgomery–Åsberg Depression Rating Scale

^g^Mini-Mental State Examination

^h^Dropouts of treatment (suicide *n* = 1; terminal illness *n* = 1; wanted to stop *n* = 3) excluded from analysis

^i^Only patients started with right unilateral electrode placement were analyzed

Mean level of IST was 60.9 ± 42.3 mC. In patients treated with BL electrode placement, mean IST was on average 50 mC higher than in those treated with RUL (95.4 ± 59.5 vs. 45.6 ± 16.8 mC; *t*-test = −4.35; d*f* = 28.9; *P* < 0.001). During the ECT course, concomitant pharmacological drugs that might influence ST were commonly used, mostly antipsychotics (64 %; *n* = 58). Of all patients, 69 % (*n* = 63) were initially treated with RUL ECT, of whom 52 % (*n* = 33) switched to BL electrode placement during the ECT course. A mean of 17.9 ± 7.4 treatments were administered during the index ECT course.

### Predictors for IST

Univariate regression analyses showed significant associations with higher IST of higher age (*P* < 0.001), higher CIRS score (*P* < 0.001), lower MMSE score at baseline (*P* = 0.002), having had previous ECT (*P* = 0.001), treatment with BL electrode placement (*P* < 0.001), lower dynamic impedance (*P* = 0.02), and lower dose of succinylcholine (*P* = 0.005) (Table 2). Multivariable regression analysis yielded higher age (β = 0.24; *P* = 0.03) and BL electrode placement (β = 0.42; *P* < 0.001) as independent predictors for higher IST. This model explained 49 % (*R* ^2^ = 0.493) of the variation in IST.

**Table 2:** Univariate and multivariate regression analyses to detect independent patient and treatment characteristics to predict initial seizure threshold (IST), and mixed analyses to detect independent predictors of the variation in seizure threshold (ST) during the course of electroconvulsive therapy (ECT)

	Univariate correlates of IST at baseline		Multivariate correlates of IST^a^ at baseline		Multivariate correlates of ST^a^ during follow-up	
	β-coefficient	*P* value	β-coefficient	*P* value	β-coefficient	*P* value
*Patient characteristics*						
Male gender	−0.043	0.682				
Age, in years	0.466	<0.001	0.238	0.033	0.279	0.009
Body mass index, in kg/m^2^	−0.156	0.143				
CIRS score^d^	0.385	<0.001	0.154	0.131	0.127	0.227
Diagnostic category, according to axis 1 of DSM-IV-R^f^						
Depressive disorder without psychotic features	−0.064	0.544				
Depressive disorder with psychotic features	0.030	0.781				
Bipolar disorder, depressive episode	0.076	0.476				
Other disorders^e^	−0.046	0.664				
Documented or clinically suspected personality disorder present, according to axis 2 of DSM-IV-R	−0.134	0.207				
MADRS^b^ score at baseline	0.082	0.450				
MMSE^c^ score at baseline	−0.331	0.002	−0.045	0.641	0.058	0.549
*Treatment characteristics*						
Had previous course(s) of ECT	0.346	0.001	0.059	0.579	−0.052	0.564
Days between last ECT and index ECT	−0.064	0.746				
Use of seizure threshold influencing concomitant pharmacotherapy						
Benzodiazepines	0.033	0.758				
Antiepileptics	0.027	0.803				
Antidepressants	0.084	0.431				
Antipsychotics	−0.112	0.289				
Electrode placement is bifrontotemporal	0.546	<0.001	0.424	<0.001	0.348	<0.001
Dynamic impedance	−0.248	0.018	−0.082	0.344	−0.107	0.062
Etomidate dose	−0.084	0.427				
Succinylcholine dose	−0.295	0.005	−0.071	0.452	−0.056	0.376

^a^Multivariate regression analysis and mixed analysis only for variables showing *P* < 0.10 at univariate analysis

^b^Montgomery–Åsberg Depression Rating Scale

^c^Mini-Mental State Examination

^d^Cumulative Illness Rating Scale

^e^Schizophrenia (*n* = 2) and malignant neuroleptic syndrome (*n* = 1)

^f^Diagnostic and Statistical Manual of Mental Disorders

### Predicting for ST change during the ECT course

**Fig. 1:**
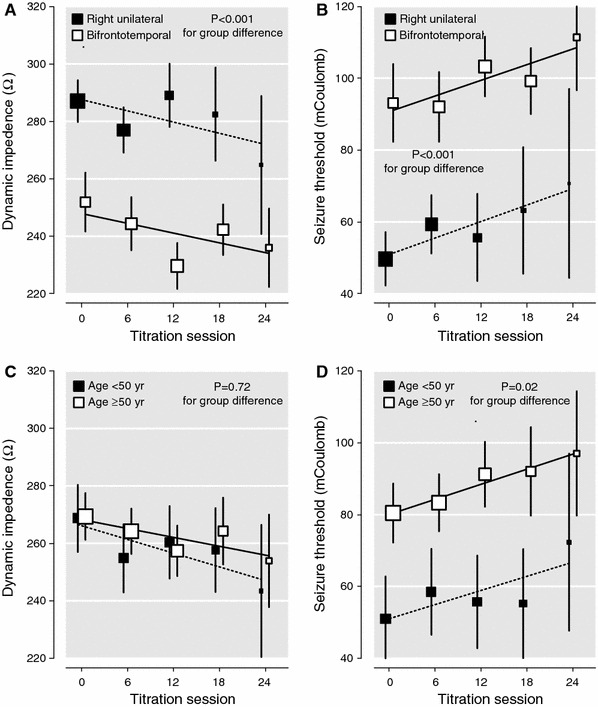
Adjusted mean values* of dynamic impedance and seizure threshold during the course of electroconvulsive therapy in patients treated with right unilateral or bifrontotemporal electrode placement (**a**, **b**) and in patients aged <50 years (*n* = 29) and ≥50 years (*n* = 62) (**c**, **d**). *Error bars represent standard errors, and *black* (*dotted)* *lines* are regression lines. The size of *each square* is proportional to the number of patients. Adjusted mean values and *P* values were calculated by multivariable regression analysis adjusted for age (for **a** and **b**), CIRS score, MMSE score at baseline, previous ECT and electrode placement (for **c** and **d**), dynamic impedance, and succinylcholine dose

ST values were similar to IST in 78 % (*n* = 67 of 86) of the patients at the ST_6_ measurement, in 60 % (*n* = 40 of 67) at ST_12_, in 44 % (*n* = 19 of 43) at ST_18_, and in 33 % (*n* = 5 of 15) at ST_24_. Table 3 presents the changes in ST over time in the RUL- and BL-treated groups. Mean ST levels increased during the course (*t* = 4.797; d*f* = 231; *P* < 0.001; see also Fig. 1b), in patients undergoing RUL and BL ECT. Six (of 86; 7 %) patients showed a lower ST_6_ than IST, as well as five (of 67; 7 %) at the ST_12_ measurement, three (of 43; 7 %) at ST_18_, and one (of 15; 7 %) at ST_24_. At the 12th session, a rise in ST with respect to IST occurred 40 % more often in BL than in RUL electrode placement (53 % [*n* = 24] vs. 14 % [*n* = 3]; Fisher’s exact test = 9.68; d*f* = 1; *P* = 0.03), with a median change of 25.2 (IQR: 0–50.4) mC in BL-treated patients and of zero (IQR: 0–0) mC in RUL-treated patients (*P* = 0.02; Table 3). At the ST_18_ measurement, the median value of ST change was also higher with BL electrode placement, but with borderline significance (*P* = 0.06); at ST_24_, the median value of ST change in BL-treated patients was higher than in the RUL group, but the difference was not significant (*P* = 0.34; Table 3).

**Table 3:** Mean values of seizure thresholds (ST), the number of patients with increased ST with respect to initial ST, and median change in ST values, during a course of electroconvulsive therapy, in patients treated with right unilateral (RUL) and bifrontotemporal (BL) electrode placements

	RUL placement				BL placement				*P* value for comparing median ST change in RUL versus BL^b^
	*n*	Mean ± SD	ST increase present (%)	Median ST change versus baseline; IQR	*n*	Mean ± SD	ST increase present (%)	Median ST change versus baseline; IQR	
*Characteristic*									
ST^a^ at initial session	63	45.6 ± 16.8			28	95.4 ± 59.5			
ST at 6th session	49	53.0 ± 25.3	9 (18.4)	0; 0–0	37	98.1 ± 77.9	11 (29.7)	0; 0–25.2	0.953
ST at 12th session	22	52.7 ± 25.7	3 (13.6)	0; 0–0	45	98.6 ± 103.0	24 (53.3)	25.2; 0–50.4	0.019
ST at 18th session	8	47.3 ± 25.0	2 (25.0)	0; 0–18.9	35	94.9 ± 55.2	22 (62.9)	25.2; 0–75.6	0.055
ST at 24th session	3	50.4 ± 0.0	2 (66.7)	25.2; 0–25.2	12	100.8 ± 52.6	8 (66.7)	50.4; 0–75.6	0.339

*SD* Standard deviation, *IQR* interquartile range

^a^In millicoulombs

^b^Mann–Whitney *U* test

Using multilevel linear models, higher age (β = 0.28; *P* = 0.009) and BL electrode placement (β = 0.35; *P* < 0.001) were independent predictors for higher ST during the course (Table 2). Mean dynamic impedance decreased during the course (*t* = −5.031; d*f* = 217; *P* < 0.001), and lower dynamic impedance showed borderline significance (β = −0.11; *P* = 0.06) for being a predictor of higher ST during the course. Figure 1 shows that, during the ECT course, adjusted mean values of dynamic impedance decreased and were significantly lower in RUL-treated patients compared with BL-treated patients (*P* < 0.001; Fig. 1a) and also that adjusted mean values of ST increased for both electrode placements and were significantly higher in patients treated with BL electrode placement compared with those treated with RUL (*P* < 0.001; Fig. 1b). Comparing patients aged <50 years and ≥50 years, no difference was found regarding adjusted mean values of dynamic impedance during the course (Fig. 1c). In patients aged ≥50 years, adjusted mean values of ST (Fig. 1d) showed a steeper increase during the ECT course than in patients aged <50 years (*P* = 0.02), illustrating the strong linear association with age as a continuous variable (Table 2).

**Fig. 2:**
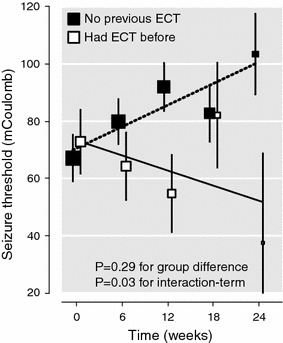
Adjusted mean values* of seizure threshold during the course of electroconvulsive therapy (ECT) in patients with no previous ECT (*n* = 63) and in patients with previous ECT course(s) (*n* = 28). *Error bars represent standard errors, and the *black* (*dotted*) *line* is the regression line. The size of *each square* is proportional to the number of patients. Adjusted mean values and the *P* value for interaction (i.e., time*previous ECT) were calculated by multivariable regression analysis adjusted for age, CIRS score, MMSE score at baseline, previous ECT, electrode placement, dynamic impedance, and succinylcholine dose

Multivariate multilevel models in which age, CIRS score at baseline, MMSE score at baseline, electrode placement, dynamic impedance, and succinylcholine dose were entered showed an independent interaction term only for having had previous ECT course(s) predicting the value of ST (*t* = 2.188; d*f* = 217; *P* = 0.03). Figure 2 shows that patients with previous ECT course(s) had less/no rise in ST during the course and that the absence of previous ECT treatment predicted a steeper rise in ST.

## Discussion

In this prospective, observational study in patients treated with ECT, higher IST was predicted by a higher age and BL electrode placement. During the ECT course, there was a rise in ST, but not in all patients. A rise in ST occurred more often in patients treated with BL electrode placement and in those having had more ECT sessions. During the course, higher age and BL electrode placement also predicted higher ST levels. At the same time, having had previous ECT course(s) was associated with an absence of ST increase and, therefore, seems to avoid any rise in ST.

### Predictors of IST and ST levels during the course

In internationally accepted guidelines, beside dose-titration methods, age-based dosing strategies are being mentioned for use in clinical practice [3]. Our study seems to justify the use of these age-based strategies, because age was an important predictor of IST and rise in ST during the course of ECT. CIRS score, dynamic impedance, lower MMSE at baseline, and succinylcholine dose were no longer independently associated with (I)ST, as they may all be critically dependent on age. Although it remains unclear why higher age is associated with higher (I)ST, several factors of morphological brain maturation related to age are hypothesized to influence ST [33]. For example, advanced age was related to both increased scalp impedance and cerebral atrophy. Furthermore, in elderly patients, less functional connectivity between brain areas (due to neuronal damage) was suggested to result in less seizure propagation [33]. Also, an ECT-induced shift of the brains inhibitory (GABA) versus excitatory (glutamate) potency has been suggested to explain ST increase during the course [22, 33]. Therefore, it is important to study these possible morphological and functional determinants prospectively [33].

In accordance with other studies [4, 9, 10, 14, 15, 18, 21, 23], we also found that BL electrode placement predicted higher ST levels during the ECT course and more often showed a rise in ST compared with RUL-treated patients. The higher ST in case of BL electrode placement might be related to differences in current pathways; that is, in BL ECT, there is a greater interelectrode distance than in RUL ECT, probably resulting in more shunting of current through the scalp which might require a higher electrical dosage to elicit seizure activity [25]. Moreover, due to the presence of hair and the difficulty of keeping two electrodes coupled to the scalp, the impedance to the passage of current might be higher and more variable with RUL than with BL electrode placement, probably resulting in a higher ST [25].

Dynamic impedance is the resistance observed between the electrodes, during the passage of the electrical stimulus, and mainly depends on electrode–skin interface (e.g., hair, conductive gel), skull thickness, and electrode placement [25]. A lower dynamic impedance is associated with BL electrode placement and with a higher IST, and the dynamic impedance was found to decrease during the ECT course [9, 10]. It has been hypothesized that dynamic impedance would decrease due to electrochemical changes in the electrode–scalp interface during the ECT course [9]. However, it is more likely that the higher electrical stimulation, due to ST increase later in the ECT course, would have resulted in lower dynamic impedance, that is, because the passage of relatively high intensity electrical stimuli resulted in much lower impedance values than the passage of lower intensity stimuli [25]. Our study supports these earlier findings, since lower dynamic impedance was associated with higher IST in univariate regression analysis, and the adjusted mean dynamic impedance values decreased during the ECT course. Also, lower dynamic impedance proved to be an independent predictor for higher ST levels during the ECT course, and dynamic impedance was lower in BL-treated patients compared with that in the RUL-treated patients.

The use of concomitant pharmacological drugs may have both increased (e.g., antiepileptics and benzodiazepines) and decreased (e.g., tricyclic antidepressants and antipsychotics) the level of IST. However, our univariate regression analyses showed no significant effect of the use of medication on IST level. This might be because, in most of our patients, medications with both ST increasing (benzodiazepines, antiepileptics) and ST decreasing (antidepressants, antipsychotics) properties were administered simultaneously, so that no specific influence could be established. Also, the anticonvulsive properties of the anesthetics may have had a stronger influence on ST, thereby masking the influence of concomitant medication, as also reported by others [5].

### Predictors of ST increase during the course

ST is reported to increase in ≤20–90 % of patients during a course of ECT, probably due to an induced shift in the brain’s inhibitory versus excitatory potency [6, 9, 11, 15, 22]. Accordingly, in the present study, mean ST levels increased during the course in ≤67 % of our patients. Although the median change in ST at the consecutive ST measurement points was higher with BL than with RUL electrode placement, in the multilevel models the interaction term of time*electrode placement was not significant.

Having undergone a previous course of ECT a considerable time before the index ECT (median: 646 days; IQR: 202–1,395 days) was independently associated with the absence of ST increase during the treatment course. Although the mean IST level of patients who underwent ECT for the first time was similar to that of patients having undergone previous ECT, first-time patients showed a rise in ST during the further ECT course, as also reported by others [14]. However, another study suggested that prior ECT course(s) resulted in higher IST in future ECT, but only in male patients [30]. In summary, previous ECT might preclude an increase in ST during the ECT course, suggesting long-term adaptation of the brain after earlier ECT. Alternatively, the association may have been confounded by the characteristic of suffering from chronic or remitting depression, being reflected in the previous ECT course; these latter patients might have a lower tendency for a rise in ST during the ECT course.

### Clinical implications

In the present study, a rise in ST occurred in a substantial proportion of patients underpinning the rationale to take age into account in ECT dosing strategies. Also, because comparison of BL and RUL ECT revealed that on average 50 mC higher IST can be expected with BL ECT, and ST increase during ECT might occur up to 40 % more often, electrode placement should be taken into account when selecting the dose of the first and subsequent ECT sessions. Furthermore, patients treated earlier with ECT might be a specific clinical group. Clinicians should be aware of the fact that previous ECT course(s) might predict ST stability during the course. This might imply that, in these patients, less electrical dose adjustments are required during the ECT course, which might also prevent cognitive side effects.

### Study limitations

Several potential limitations need to be addressed. First, for ECT, ST is determined by several physical stimulus characteristics (e.g., pulse width, frequency, current strength) and by the minimum seizure duration selected to be sufficient in the titration procedure. In earlier studies, different settings and definitions have been used [1, 29], thus hampering comparison of our estimated ST levels with those of others. Furthermore, our ECT device controls charge delivery by frequency, stimulus train duration, and setting of the pulse width, resulting in changes of these parameters when delivering higher charges [1]. This might have influenced the measurements of IST and ST levels, although the changes were the same for those patients who needed higher charges. Second, our ST levels were determined by a somewhat crude method, because the titration protocol consisted of steps of ≥25 mC. Using an ECT device that offers the possibility to increase the dose with (very low) steps might have refined the titration procedure, but this method would probably be limited due to the maximum length of anesthesia and muscle paralysis. Furthermore, we used an age-based adjustment in our titration protocol [10], which may have led to some bias and circularity, as such adjustment may explain (in part) the higher ST levels in older patients. Finally, almost all of our patients used concomitant medication during the ECT procedure.

In conclusion, in 91 prospectively examined, mostly depressed and older patients treated with ECT, age and electrode placement independently predicted IST and ST levels during the course. Some patients showed a persistent rise in ST at the consecutive measurement points. However, having had previous ECT course(s) predicted an absence of ST increase during the ECT course, independent of age and electrode placement. Future studies should explore whether or not these factors adversely affect treatment response.
